# Profiling the impact of different tau species on glial cell biology

**DOI:** 10.3389/fcell.2025.1622138

**Published:** 2025-07-17

**Authors:** Ines Arribas Gomez, Yan Yan, Meredith T. Lilley, Yunfei Chen, Lillian M. Daughrity, Ana Moreno Arnas, Ji Shi, Jennifer M. Kachergus, E. Aubrey Thompson, Karen Jansen-West, Casey N. Cook

**Affiliations:** ^1^ Department of Neuroscience, Mayo Clinic, Jacksonville, FL, United States; ^2^ Division of Gastroenterology and Hepatology, Mayo Clinic, Jacksonville, FL, United States; ^3^ Neuroscience Graduate Program, Mayo Graduate School, Mayo Clinic College of Medicine, Jacksonville, FL, United States; ^4^ Department of Cancer Biology, Mayo Clinic, Jacksonville, FL, United States; ^5^ Department of Molecular Medicine, University of South Florida, Tampa, FL, United States

**Keywords:** tauopathy, glial cell profiling, NanoString, P301L, A152T, solubility, oligomer

## Abstract

**Introduction:**

Tauopathies are a heterogeneous group of neurodegenerative disorders characterized by abnormal tau protein accumulation in neuronal and/or glial cells. Different pathogenic tau mutations result in distinct patterns of tau deposition, yet the differential effects of these tau species on glial cell biology are poorly understood. This study examines glial cell function in response to two distinct tau variants: P301L (promoting insoluble/fibrillar tau) and A152T (favoring soluble/oligomeric tau).

**Methods:**

We used adeno-associated virus to express human tau containing either the P301L or A152T mutation and delivered to the brain by intracerebroventricular injection on postnatal day 0. At 3 months of age, we used the nCounter mouse glial profiling panel to measure expression of 770 genes involved in glial cell biology in the brain. Differential expression and pathway analysis, as well as cell type profiling were performed to assess how glial cell signatures in P301L-AAV and A152T-AAV mice differ in comparison to the control group (GFP-AAV injected mice).

**Results:**

P301L-AAV and A152T-AAV mice exhibited both common and distinct changes in their glial gene expression profiles. P301L-AAV mice showed a pronounced microglial inflammatory response with upregulation of microglial activation markers (Clec7a, Cst7, Gpr84) and inflammatory mediators (Ccl3, Nlrp3). A152T-AAV mice demonstrated a more prominent astrocytic response with upregulation of astrocyte-specific genes (Gdpd2, Ggta1, Aqp4, Fbln5). In addition, only A152T-AAV mice exhibited coordinated impairment in glucose metabolism, mitochondrial function, calcium signaling, protein clearance, and increased apoptotic signaling.

**Discussion:**

Our findings reveal that different patterns of tau accumulation elicit fundamentally distinct glial responses. Insoluble tau deposition (P301L) primarily triggers microglial inflammatory pathways without substantial metabolic disruption, suggesting a direct response to tau fibrils. In contrast, soluble tau species (A152T) impact multiple cellular mechanisms simultaneously, including metabolic function, calcium homeostasis, and phagocytosis, potentially explaining the neuronal loss previously observed in this model. These distinct cellular signatures expand our understanding of how tau contributes to neurodegeneration and may inform more targeted therapeutic strategies based on predominant patterns of tau accumulation in different tauopathies.

## 1 Introduction

Tauopathies are a heterogeneous group of neurodegenerative disorders that includes corticobasal degeneration (CBD), progressive supranuclear palsy (PSP), Pick’s disease (PiD), Alzheimer’s disease (AD), and chronic traumatic encephalopathy (Devi, 2023). Neuropathologically, tauopathies are characterized by abnormal deposition of the tau protein within either neuronal and/or glial cells. While tau normally functions as a microtubule-associated protein that is crucial for maintaining cell structure and trafficking, the discovery of pathogenic mutations in the tau gene (MAPT) (Hutton et al., 1998; Poorkaj et al., 1998; Spillantini et al., 1998) indicated that tau dysfunction alone is sufficient to cause disease. Additional support for this idea is provided by the neurodegenerative phenotype observed in mice expressing human tau protein containing the P301L mutation (Lewis et al., 2000), one of the first pathogenic MAPT mutations identified in patients with familial frontotemporal dementia (FTD) (Hutton et al., 1998). In particular, transgenic P301L mice exhibited tau aggregation and neurofibrillary tangle formation in an age- and gene-dosage dependent manner, as well as neurodegeneration and behavioral defects (Lewis et al., 2000).

In contrast to P301L and other pathogenic tau mutations, the A152T genetic variant is associated with risk of either AD or frontotemporal spectrum disorders, such as PSP and CBD (Coppola et al., 2012), as well as dementia with Lewy bodies (Labbe et al., 2015). Although the mechanism by which the A152T variant modulates disease risk remains unclear, the A152T mutation has been shown to decrease the ability of tau to bind microtubules, and also favor tau oligomer formation while impeding filament assembly and aggregation (Coppola et al., 2012). As such, given the distinct molecular effects on tau, comparing the resulting phenotype and pattern of tau deposition in animals expressing either P301L or A152T mutant tau provides a unique opportunity to illuminate both mechanisms of disease pathogenesis and factors that determine and modulate disease risk in tauopathy. In order to do so, we previously generated adeno-associated virus (AAV) driving expression of human tau containing either the P301L or A152T mutation, and delivered to the brain by intracerebroventricular injection on postnatal day 0 (Cook et al., 2015; Carlomagno et al., 2019). At 3 months of age, we found that P301L-AAV mice exhibited accumulation of hyperphosphorylated tau primarily in the insoluble fraction, while tau deposition in A152T-AAV mice was localized to the soluble fraction despite robust hyperphosphorylation (Carlomagno et al., 2019). The restriction of A152T-expressing tau to the soluble fraction is also consistent with observations in a transgenic mouse model (Maeda et al., 2024), suggesting this variant increases disease risk through promoting tau oligomerization but preventing aggregation.

Building on this work, we sought to examine how glial cell function is differentially affected by the distinct patterns of tau deposition observed in the P301L-AAV and A152T-AAV models. We therefore utilized a mouse glial profiling panel from Nanostring to measure the expression of 770 genes involved in glial cell biology. Notably, this panel was previously used in human patients with frontotemporal lobar degeneration with tau pathology (FTLD-tau), which revealed significant glial dysregulation in FTLD-tau compared to controls, including alterations in multiple astrocyte-, microglia-, and oligodendrocyte-related pathways (Ferrer et al., 2014). In the current study, we found that these different patterns of tau accumulation are associated with distinct glial responses, but also differentially affect metabolic function, as well as phagocytosis and apoptosis.

## 2 Materials and methods

### 2.1 Viral vector construction and AAV production

The coding sequences for EGFP, TauA152T (C-terminal V5 tag), and TauP301L (C-terminal V5 tag) were subcloned into an AAV vector and sequence-verified using ABI3730 with Big Dye chemistry (Applied Biosystems, Foster City, CA). AAV vectors containing EGFP, TauA152T, and TauP301L under the control of the cytomegalovirus enhancer/chicken β-actin promoter, as well as a woodchuck post-transcriptional regulatory element and the bovine growth hormone polyA, were cotransfected with AAV helper plasmids into HEK293T cells. Cells were harvested and lysed in the presence of 0.5% sodium deoxycholate and 50 U/mL Benzonase (Sigma, St. Louis, MO) by freeze thawing 48 h post-transfection, and the virus was isolated using a discontinuous iodixanol gradient. Quantitative PCR was used to measure the genomic titer of each virus.

### 2.2 Intracerebroventricular injections

All animal procedures were approved by the Mayo Institutional Animal Care and Use Committee (IACUC) and are in accordance with the National Institutes of Health Guide for the Care and Use of Laboratory Animals (NIH Publications No. 80-23, revised 1996). Intracerebroventricular (ICV) injections with either TauA152T-AAV, TauP301L-AAV or EGFP-AAV were performed into mouse pups on postnatal day 0 (2.7E + 10 viral particles/ventricle; 2 μL/ventricle) as described (Chakrabarty et al., 2013). Briefly, newborn mice were cryoanesthetized and placed on a cold metal plate. A 10 μL Hamilton syringe with a 30-gauge needle was used to pierce the skull just posterior to bregma and 2 mm lateral to the midline, and 2 μL of AAV was injected into each of the lateral ventricles. Mice were euthanized by CO_2_ inhalation at 3 months of age. The brain was immediately removed and frozen for RNA extraction and analysis. Please see Supplementary Table S1 for sample information.

### 2.3 Generation of mouse brain lysates and RNA extraction

Half brains were weighed and homogenized in 5× volume of TE buffer [50 mM Tris base (pH 7.4), 50 mM NaCl, 1 mM EDTA, 1 mM PMSF, 1× protease and phosphatase inhibitors]. For RNA isolation, 70 μL of homogenate was added to 210 μL Trizol LS (Life Technologies, Carlsbad, CA), and the mixture frozen at −80°C until extraction. Total RNA was isolated from brain tissue using the Direct-zol RNA Miniprep kit (Zymo Research, Irvine, CA) according to manufacturer’s instructions with in-column DNase I treatment. RNA concentrations and A260/280 ratios were measured using a Nanodrop.

### 2.4 NanoString nCounter glial profiling panel and statistical analysis

The nCounter mouse glial profiling panel (NanoString, USA) was used to quantify expression in mouse RNA samples, with data analyzed using an nCounter FLEX Analysis System (NanoString, USA). Raw data were imported into nSolver 4.0 and analyzed with the nCounter advanced analysis paired with R (version 3.3.2). Following normalization and quality control, data was analyzed to assess differential expression, as well as perform pathway and cell type profiling analyses. Mice were age-matched and all samples were included on one cartridge, eliminating the need to control for age or batch effects. To determine whether differences between GFP-AAV, P301L-AAV, and A152T-AAV animals were statistically significant, differences between groups were assessed using 1-way ANOVA followed by a Tukey’s post-hoc test for multiple comparisons. All statistical analyses were performed in R and GraphPad Prism, and data are presented as mean ± SEM, with p < 0.05 considered statistically significant.

## 3 Results

### 3.1 Evaluating the impact of tau accumulation on glial cell biology

**FIGURE 1:**
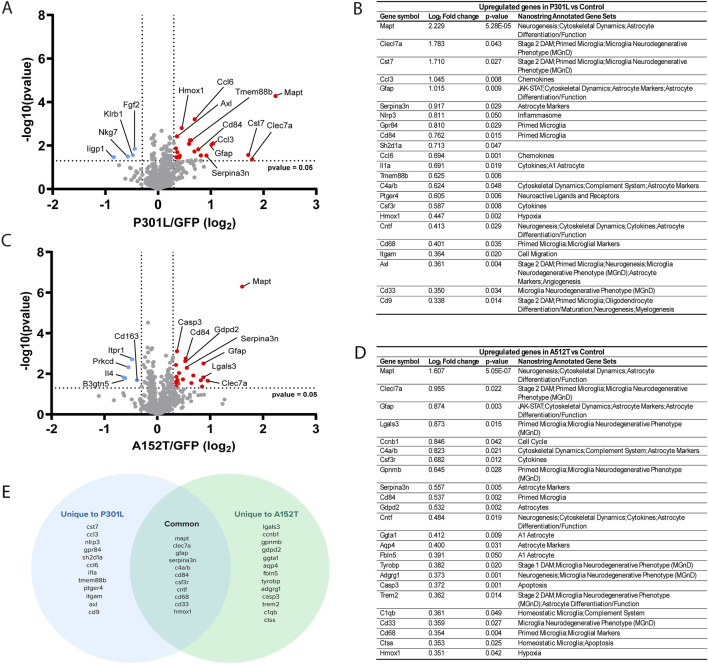
Evaluating the impact of tau accumulation on glial cell biology. **(A)** Volcano plot depicting differential gene expression in P301L-AAV mice compared to GFP-AAV control mice. **(B)** Table of significantly upregulated genes in P301L-AAV mice displaying Log_2_ fold change, p-value, and their association with Nanostring-annotated functional gene sets. **(C)** Volcano plot depicting differential gene expression in A152T-AAV mice compared to GFP-AAV control mice. **(D)** Table of significantly upregulated genes in A152T-AAV mice displaying Log_2_ fold change, p-value, and their association with Nanostring-annotated functional gene sets. **(E)** Venn diagram illustrating common and uniquely upregulated genes between P301L-AAV and A152T-AAV mice. In volcano plots **(A,C)**, red dots represent significantly upregulated genes while blue dots represent significantly downregulated genes. Dotted lines indicate statistical significance threshold (p-value <0.05) and expression threshold (Log_2_ fold change > |0.3|).

We previously observed that mice expressing the P301L mutation exhibit accumulation of insoluble hyperphosphorylated tau and microgliosis in the absence of neuronal loss (Cook et al., 2015; Carlomagno et al., 2019). Conversely, accumulation of soluble hyperphosphorylated tau, astrocytosis and neuronal loss are characteristic of mice expressing A152T tau (Carlomagno et al., 2019). To illuminate how these different patterns of tau deposition differentially impact glial cell biology, we performed a comprehensive analysis using the nCounter glial profiling panel to simultaneously quantify mRNA expression of 770 genes (Supplementary Table S2). Differential expression analysis revealed that P301L-AAV (Figures 1A,B) and A152T-AAV mice (Figures 1C,D) exhibit distinct changes compared to the control group (GFP-AAV).

In P301L-AAV mice, the expression of 23 genes was upregulated (Figures 1A,B) and four downregulated (Figures 1A,B). Among the top 10 upregulated genes were multiple markers associated with inflammatory microglia, including *Clec7a*, *Cst7*, *Gpr84*, and *Cd84*, suggesting a strong microglial response associated with insoluble tau deposition in P301L-AAV mice. In addition, genes linked with astrocyte activation (*Gfap*, *Serpina3n*) and inflammatory signaling (*Ccl3*, *Nlrp3*, *Sh2d1a*) were also significantly upregulated in P301L-AAV mice, along with *Mapt* itself. Downregulated genes include the immune response regulator *Klrb1*, genes involved in interferon signaling (*Iigp1* and *Nkg7*), and *Fgf2* involved in cellular growth signaling.

In A152T-AAV mice, the expression of 24 genes was upregulated and four downregulated compared to the control group (Figures 1C,D). Among the top upregulated genes, similar to P301L-AAV mice we observed increased expression of microglial activation markers (*Clec7a*, *Lgals3*, *Gpnmb*, *Cd84*) and the complement component *C4a/b*, in addition to astrocyte-related genes (*Gfap*, *Serpina3n*) in A152T-AAV mice. Additional astrocyte markers that were unchanged in P301L-AAV mice were increased in A152T-AAV mice, including *Gdpd2*, *Ggta1*, *Aqp4*, and *Fbln5*. Downregulated genes in A152T-AAV mice include genes involved in T cell signaling and cytokine responses (*Il4*), glycolipid metabolism (*B3gnt5*), phagocytosis and neurotrophin signaling (*Prkcd*), calcium signaling (*Itpr1*), and immune cell function (*Cd163*).

While both models showed increased *Mapt* expression (providing experimental validation of the panel) as well as upregulation of several common genes involved in neuroinflammation, including *Clec7a*, *Gfap*, *Serpina3n*, *Cd84*, and *C4a/b*, each tau mutation induced unique gene expression patterns (Figure 1E). In particular, an upregulation of genes associated with inflammatory microglial responses (*Ccl3*, *Nlrp3*, *Gpr84*, *Sh2d1a*), chemokines (*Ccl6*, *Ccl3*), and cytokine signaling (*Il1a*, *Tmem88b*) were only observed in P301L-AAV mice, suggesting a stronger microglial inflammatory phenotype. In contrast, A152T-AAV mice uniquely upregulated genes related to astrocyte function (*Gdpd2*, *Ggta1*, *Aqp4*, *Fbln5*), apoptotic signaling (*Casp3*), and homeostatic microglial functions (*C1qb*, *Ctss*), indicating a more prominent astrocytic response.

### 3.2 Pathway analysis reveals distinct molecular signatures in mice expressing P301L and A152T mutant tau

**FIGURE 2:**
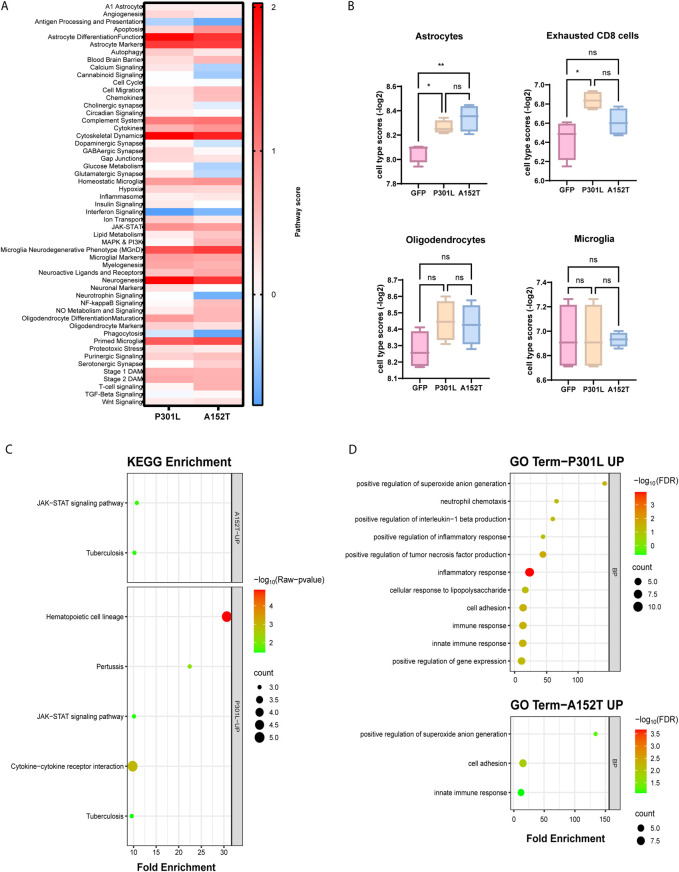
Pathway analysis reveals distinct molecular signatures in mice expressing P301L and A152T mutant tau. **(A)** Heatmap displaying pathway scores between P301L-AAV or A152T-AAV mice compared to GFP-AAV control mice. Color intensity represents the magnitude of difference with the control group, with red indicating increased pathway activity and blue indicating decreased pathway activity in the tau-AAV groups. **(B)** Cell type-specific gene expression signatures quantified by NanoString cell type scores for astrocytes, exhausted CD8 T cells, oligodendrocytes and microglia in GFP-AAV, P301L-AAV, and A152T-AAV mice. Statistical significance was determined using one-way ANOVA, with significance indicated by *p < 0.05, **p < 0.01 and ns = not significant. **(C)** KEGG pathway enrichment analysis of upregulated genes in P301L-AAV and A152T-AAV mice. Circle size corresponds to gene count within each pathway and color intensity represents statistical significance [−log_10_(p-value)]. **(D)** Gene Ontology (GO) term enrichment analysis of upregulated genes in P301L-AAV (upper panel) and A152T-AAV (lower panel) mice. Circle size indicates gene count for each term and color intensity represents statistical significance [−log_10_(FDR)].

To better understand the biological significance of differentially expressed genes, we performed pathway-level analyses comparing both tau-AAV models to GFP-AAV controls (Figure 2A, Supplementary Table S3). Both models showed strong activation of multiple astrocyte-related pathways, including astrocyte differentiation/function and markers, consistent with the upregulation of glial fibrillary acidic protein (*Gfap*) and Serpin Family A Member 3N (*Serpina3n*). Similarly, microglial pathways, including the microglial neurodegenerative phenotype (MGnD) and microglial markers, showed marked increases in both P301L-AAV and A152T-AAV mice, reflecting the elevated expression of microglial genes like *Clec7a*, *Cd84* and *Cd68*. While both models also exhibited decreased activity in antigen processing and presentation and interferon signaling pathways, the complement system and cytokine pathways were increased, aligning with the increased expression of inflammatory mediators like *C4a/b*. Moreover, consistent with the significant increase in *Mapt* expression in both models combined with tau’s known role in cytoskeletal regulation, cytoskeletal dynamics was one of the most upregulated pathways in both tau-AAV groups. Both models also showed activation of JAK-STAT signaling, while neurotrophin signaling and phagocytosis were most reduced in A152T-AAV mice.

In addition to examining pathway scores, we also performed cell type profiling to understand how distinct patterns of tau deposition may differentially impact cellular populations (Supplementary Table S4). Notably, astrocyte scores were significantly increased in both tau-AAV models compared to the GFP-AAV control group (Figure 2B), which may indicate that accumulation of hyperphosphorylated tau independent of changes in solubility may be linked with astrocytosis. Exhausted CD8 T cell scores were also significantly increased but only in P301L-AAV mice (Figure 2B), which suggests a relationship with insoluble tau accumulation. Finally, despite activation of both oligodendrocyte- and microglial-related pathways in both tau models (Figure 2A), oligodendrocyte and microglia cell type scores showed no significant differences between groups (Figure 2B).

To further illuminate functional differences between tau models, we performed pathway enrichment analysis, which revealed both common and model-specific changes. In particular, while both tau-AAV models showed enrichment in JAK-STAT signaling, hematopoietic cell lineage and cytokine-cytokine receptor interaction pathways were enriched only in P301L-AAV mice (Figure 2C), consistent with the elevated expression of Ccl3, Ccl6, and Cst7. Gene Ontology (GO) enrichment analysis revealed similar changes in both tau-AAV models, including significant enrichment in the biological processes (BP) positive regulation of superoxide anion generation’, “cell adhesion” and “innate immune response” (Figure 2D). However, additional terms were enriched only in P301L-AAV mice, including “neutrophil chemotaxis”, “inflammatory response”, “cellular response to lipopolysaccharide” and “positive regulation of tumor necrosis factor production”, collectively reflecting a pronounced inflammatory signature associated with insoluble tau deposition characteristic of the P301L-AAV model.

### 3.3 Tau deposition leads to transcriptional alterations in CNS-resident glial cells

**FIGURE 3:**
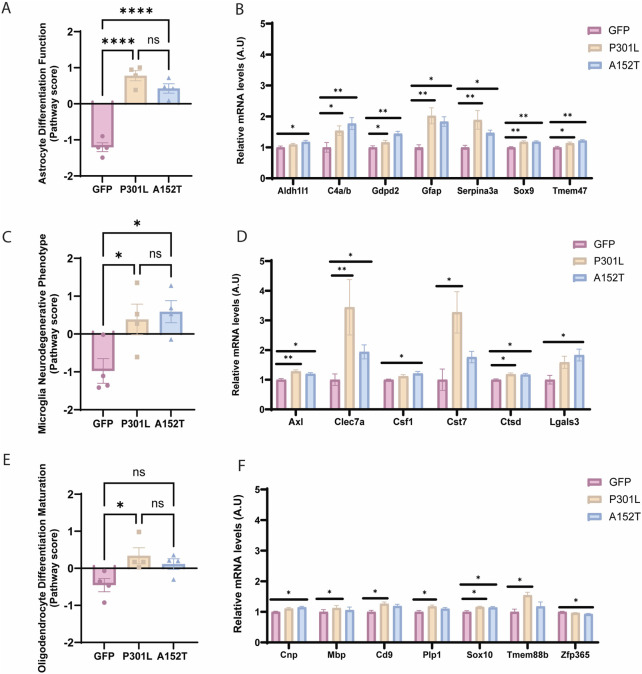
Tau deposition leads to transcriptional alterations in CNS-resident glial cells. Left panels show pathway scores and right panels show normalized mRNA levels (A.U.) of key genes for GFP-AAV (pink), P301L-AAV (beige), and A152T-AAV (blue) groups. **(A)** Astrocyte differentiation and function score. **(B)** Astrocyte-related genes: *Aldh1l1, C4a/b, Gdpd2, Gfap, Serpina3n, Sox9*, and *Tmem47*. **(C)** Microglial neurodegenerative phenotype (MGnD) score. **(D)** Microglia-related genes: *Axl, Clec7a, Csf1, Cst7, Ctsd* and *Lgals3*. **(E)** Oligodendrocyte differentiation and maturation score. **(F)** Oligodendrocyte-related genes: *Cnp*, *Mbp*, *Cd9*, *Plp1*, *Sox10*, *Tmem88b*, and *Zfp365*. All mRNA expression data are normalized to GFP-AAV control levels. Statistical significance: *p < 0.05, **p < 0.01, ***p < 0.0001, and ns = not significant. A.U. = arbitrary units.

Both tau-AAV models were associated with significant differences in astrocyte differentiation and function compared to the GFP-AAV control group (Figure 3A), reflected in elevated astrocyte cell scores (Figure 2B). Although *Sox9* and *Aldh1l1* were increased in both P301L-AAV and A152T-AAV mice (Figure 3B) suggesting a more pronounced maturation profile (Sun et al., 2017; Yang et al., 2011), a key distinguishing pattern emerged in astrocyte activation markers. In particular, the classic reactive astrocyte markers *Gfap* and *Serpina3n* (Eng and Ghirnikar, 1994; Zamanian et al., 2012) exhibited higher expression in P301L-AAV mice (Figure 3B), implicating a relationship with insoluble tau accumulation. Moreover, while both models were associated with increased *C4a/b* expression, A152T-AAV mice were more prominently affected (Figure 3B).

Regarding microglia-associated impacts, despite the fact that overall microglial cell scores remained unchanged (Figure 2D), the microglial neurodegenerative phenotype (MGnD) was increased in both tau-AAV models compared to control (Figure 3C). Looking at individual targets, *Axl* and *Ctsd* were increased in both tau-AAV models (Figure 3D), which may implicate a compensatory upregulation in lysosomal activity to facilitate debris clearance and damage sensing capabilities (Kenessey et al., 1997; Kim et al., 2007; Fou et al., 2016). Of interest, *Lgals3* (galectin-3) was only upregulated in A152T-AAV mice (Figure 3D), which is intriguing given that galectin-3 has been shown to bind hyperphosphorylated tau and augment tau aggregation (Siew et al., 2024). In addition, the increase in *Clec7a* and *Cst7* expression was most robust in P301L-AAV mice, potentially indicative of a more pronounced inflammatory phenotype (Sala Frigerio et al., 2019; Keren-Shaul et al., 2017).

Oligodendrocyte differentiation and maturation was also increased in P301L-AAV mice compared to control (Figure 3E), with broad upregulation of several targets including *Sox10*, *Mbp*, *Cd9*, *Plp1*, and *Tmem88b* (Figure 3F), suggesting enhanced myelination potential (Deber and Reynolds, 1991; Galiano et al., 2006; Zeis et al., 2016; Rubinstein et al., 1996; Klugmann et al., 1997; Griffiths et al., 1998; Luders et al., 2017). While the oligodendrocyte lineage marker *Sox10* was also increased in A152T-AAV mice relative to control, model-specific changes include a modest increase in *Cnp* and slight reduction in *Zfp365* (Figure 3F), which may be indicative of subtle changes in oligodendrocyte differentiation patterns (Fan et al., 2023; Shimizu et al., 2014; Tohyama et al., 2015).

### 3.4 Tauopathy-associated changes in metabolic and mitochondrial function

**FIGURE 4:**
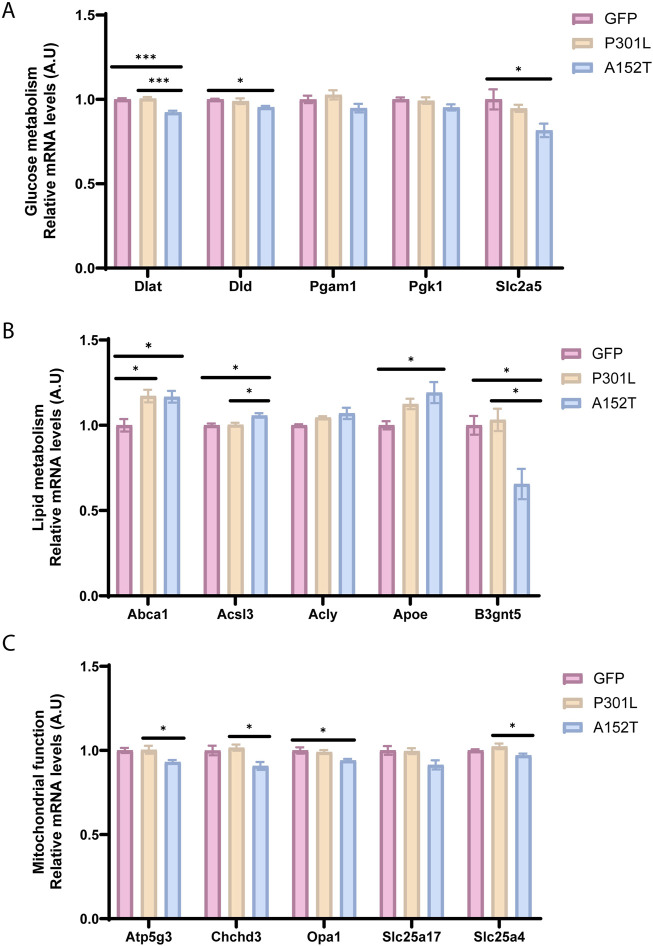
Tauopathy-associated changes in metabolic and mitochondrial function. Relative mRNA levels of key metabolic genes for GFP-AAV (pink), P301L-AAV (beige), and A152T-AAV (blue) groups. **(A)** mRNA levels of glucose metabolism-related genes: *Dlat*, *Dld*, *Pgam1*, *Pgk1*, and *Slc2a5* **(B)** mRNA levels of lipid metabolism-related genes: *Abca1*, *Acsl3*, *Acly*, *Apoe*, and *B3gnt5* **(C)** mRNA levels of mitochondrial markers: *Atp5g3*, *Chchd3*, *Opa1*, *Slc25a17*, and *Slc25a4*. All mRNA expression data are normalized to GFP-AAV control levels. Statistical significance: *p < 0.05, ***p < 0.001. A.U. = arbitrary units.

Our analysis revealed both common and model-specific changes in glucose and lipid metabolism, as well as mitochondrial function. In particular, changes in expression of genes associated with glucose metabolism were observed in A152T-AAV but not P301L-AAV mice (Figures 4A,B), including the simultaneous downregulation of both *Dlat* and *Dld* - key enzymes bridging glycolysis and the TCA cycle. Expression of the glucose transporter *Slc2a5* (Glut5) was also decreased in A152T-AAV mice (Figure 4B). This coordinated suppression suggests a fundamental disruption in cellular energy production in the A152T-AAV model.

As lipid metabolism plays a crucial role in brain function and has been increasingly recognized as a key factor that is dysregulated in neurodegenerative diseases (Estes et al., 2021), it is intriguing that expression of *Abca1*, which is essential for lipid homeostasis in neurons and glia (Dean et al., 2001; Tachikawa et al., 2005; Hirsch-Reinshagen et al., 2004; Wellington et al., 2002), showed elevated expression in both P301L and A152T groups (Figure 4B). However, only A152T-AAV mice exhibited an increased *Apoe* and decreased *B3gnt5* expression (Figure 4B), potentially implicating alterations in glycolipid metabolism in this model.

Building on alterations within metabolic pathways, we next examined expression of mitochondrial genes in both tau models (Figure 4C). Of note, only A152T-AAV mice exhibited a reduction in *Atp5g3* expression (Figure 4C), which is essential for ATP synthesis during oxidative phosphorylation (Huang et al., 2013). In addition to *Atp5g3*, expression of other mitochondrial genes was also decreased in A152T but not P301L-AAV mice, including *Chchd3* and *Opa1* (Figure 4C). Of particular relevance, Chchd3 plays a critical role in maintaining mitochondrial crista architecture (Schauble et al., 2007; Dreger et al., 2005) and also regulates Opa1 (Darshi et al., 2011), which is a dynamin-related GTPase that is essential for mitochondrial fusion and cristae maintenance (MacVicar and Langer, 2016). Overall, these coordinated changes across glucose metabolism, lipid processing, and mitochondrial function pathways suggest A152T mutant tau expression is associated with widespread metabolic reprogramming that is distinct from the phenotype associated with P301L mutant tau expression.

### 3.5 A152T mutant tau expression leads to changes in apoptotic, calcium signaling, and phagocytic pathways

**FIGURE 5:**
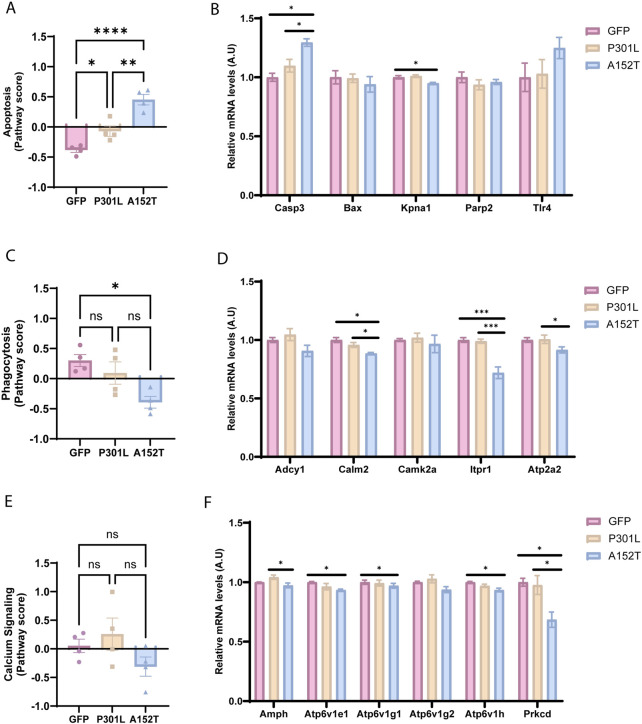
A152T mutant tau expression leads to changes in apoptotic, calcium signaling, and phagocytic pathways. Left panels show normalized pathway scores and right panels show normalized mRNA levels of key genes for GFP-AAV (pink), P301L-AAV (beige), and A152T-AAV (blue) groups. **(A)** Apoptosis pathway score **(B)** mRNA levels of apoptosis-related genes: *Casp3, Bax, Kpna1, Parp2*, and *Tlr4* **(C)** Calcium signaling score **(D)** mRNA levels of calcium signaling-related genes: *Adcy1*, *Calm2*, *Camk2a*, *Itpr1* and *Atp2a2* **(E)** Phagocytosis pathway score **(F)** mRNA levels of phagocytosis-related genes: *Amph*, *Atp6v1e1*, *Atp6v1g1*, *Atp6v1g2*, *Atp6v1h*, and *Prkcd*. All mRNA expression data are normalized to GFP-AAV control levels. Statistical significance: *p < 0.05, **p < 0.01, ***p < 0.001, ****p < 0.0001. A.U. = arbitrary units.

In addition to metabolic alterations, A152T-AAV mice also exhibited more dramatic alterations than P301L-AAV mice across multiple cellular processes. In particular, apoptosis was significantly upregulated in A152T-AAV mice compared to both GFP-AAV and P301L-AAV groups (Figure 5A), which is likely driven by a robust increase in caspase 3 (*Casp3*) expression (Figure 5B). Increased expression of toll-like receptor 4 (*Tlr4*) in A152T-AAV mice was also trending but not significant, while levels of the nuclear import adaptor karyopherin-α1 (*Kpna1*) were significantly reduced by mutant A152T tau expression (Figure 5B). In contrast, levels of the apoptosis regulator bcl-2-like protein 4 (*Bax*) and poly (ADP-ribose) polymerase 2 (*Parp2*) expression were unchanged in either model.

Calcium signaling (Figures 5C,D) and phagocytosis pathways (Figures 5E,F) also showed mutation-specific effects, with alterations detected within the A152T-AAV but not P301L-AAV models. As key regulators of calcium signaling in the cell, expression of calmodulin 2 (*Calm2*), inositol 1,4,5-triphosphate receptor (*Itpr1*), and ATPase sarcoplasmic/endoplasmic reticulum Ca2+ transporting 2 (*Atp2a2*) were all reduced in A152T-AAV mice (Figure 5D). As the phagocytosis pathway is also significantly reduced in A152T animals (Figure 5E), it is noteworthy that several subunits of the V-ATPase complex (*Atp6v1e1*, *Atp6v1g1*, *Atp6v1h*) showed coordinated downregulation, implicating an impairment of lysosomal function (Colacurcio and Nixon, 2016). Additionally, reduced expression of protein kinase C delta (*Prkcd*) in A152T mice suggests mitophagy may also be perturbed (Munson et al., 2021). Collectively, these changes suggest that apoptotic signaling is elevated while cellular calcium homeostasis and phagocytosis/lysosomal function is inhibited by the accumulation of soluble hyperphosphorylated tau species in A152T mutant tau expressing mice.

## 4 Discussion

In this study, we utilized two different tau mutations as tools to drive distinct patterns of tau accumulation - insoluble/fibrillar hyperphosphorylated tau in P301L-AAV mice versus soluble/oligomeric hyperphosphorylated tau in A152T-AAV mice - and examined their differential impact on glial cell biology. Our gene expression analysis revealed that these distinct patterns of tau deposition elicit unique glial signatures with implications for understanding tauopathy pathogenesis. In particular, differential expression analysis demonstrated that while both forms of pathological tau trigger neuroinflammatory responses, the nature of these responses differ markedly depending on tau’s aggregation state. Insoluble tau accumulation in P301L-AAV mice primarily elicited a pronounced microglial inflammatory response, characterized by upregulation of inflammatory mediators (*Ccl3*, *Nlrp3*, *Gpr84*) and cytokine signaling pathways (Figures 1A,B). This aligns with previous observations in both human tauopathies and animal models where fibrillar tau accumulation correlates with microglial activation and inflammatory cytokine production (Bhaskar et al., 2010; Ising et al., 2019; Hopp et al., 2018). The enrichment of pathways related to neutrophil chemotaxis, inflammatory response, and TNF production specifically in P301L-AAV mice (Figure 2D) further support a model where fibrillar tau accumulation drives a robust neuroinflammatory phenotype.

In contrast, soluble hyperphosphorylated tau accumulation in A152T-AAV mice was associated with a pronounced astrocytic response, evidenced by unique upregulation of multiple astrocyte-specific genes (*Gdpd2*, *Ggta1*, *Aqp4, Fbln5*) (Figures 1C,D) and higher astrocyte cell type scores (Figure 2B). While both tau models showed upregulation of classic reactive astrocyte markers *Gfap* and *Serpina3n* (Figure 1E), expression levels were notably higher in P301L-AAV mice, suggesting that fibrillar tau accumulation may trigger a stronger classical astrocyte activation profile despite the broader astrocytic response in A152T-AAV mice. This pattern is consistent with previous reports of prominent astrocytosis in transgenic A152T mouse models (Maeda et al., 2016; Sydow et al., 2016). Moreover, the simultaneous upregulation of *C1qb* and *Ctss* (Figures 1C,D), markers of homeostatic microglia (Sala Frigerio et al., 2019; Sousa et al., 2018), may be indicative of a coordinated glial response focused more on maintaining tissue homeostasis than driving inflammation in A152T-AAV mice.

Further examination of specific glial populations revealed that the microglial neurodegenerative phenotype (MGnD) was activated in both models (Figure 3C) despite unchanged overall microglial cell scores (Figure 2B). This finding highlights the importance of phenotypic characterization beyond simple abundance metrics. In addition, the robust upregulation specifically in P301L-AAV mice of C-type lectin domain containing 7A (*Clec7a*) and cystatin F (*Cst7*) (Figure 3D), both well-established markers of disease-associated microglia (DAM)/MGnD (Krasemann et al., 2017; Butovsky and Weiner, 2018; Daniels et al., 2023), suggests a critical role for fibrillar tau deposition in acquisition of the DAM/MGnD signature. P301L-AAV mice also exhibited elevated activation of the oligodendrocyte differentiation and maturation pathway (Figures 3E,F). The coordinated upregulation of myelination-associated transcripts (*Sox10*, *Mbp*, *Cd9*, *Plp1*) suggests that fibrillar tau may promote expression of genes that regulate oligodendrocyte development, at least at this early timepoint. Notably, demyelination is a consistent feature of AD (Benitez et al., 2014; Gao et al., 2011; Reisberg et al., 1999) and has been observed in patients with mild cognitive impairment (MCI) (Bouhrara et al., 2018), implicating dysregulation of the oligodendrocyte cell population even at early stages of disease. Recently, multiple groups have identified transcriptional alterations in myelination networks as a key feature of AD (Allen et al., 2018; Grubman et al., 2019; Mathys et al., 2019; McKenzie et al., 2017; Zhou et al., 2020), further underscoring the need to elucidate disease-related alterations in cells of the oligodendrocyte lineage to understand their role in the pathophysiology of AD and related disorders. In light of this published work, the current findings may indicate that upregulation of genes associated with oligodendrocyte differentiation/maturation in young P301L-AAV mice is a compensatory response to loss of white matter integrity or myelination defects. Future studies are needed to illuminate the mechanistic relationship between tau pathology and myelination abnormalities *in vivo*.

In addition to glial phenotyping, evaluation of metabolic pathways also uncovered a striking distinction between tau models. In particular, we observed a coordinated suppression of glucose metabolism genes (*Dlat*, *Dld*, *Slc2a5*) alongside reductions in mitochondrial-associated genes (*Atp5g3*, *Chchd3*, *Opa1*) in A152T-AAV mice (Figures 4A,C), suggesting that fundamental energy production deficits are associated with the accumulation of soluble tau species. These findings align with emerging evidence that soluble tau oligomers may be especially toxic to mitochondria (Lasagna-Reeves et al., 2011; Szabo et al., 2020; Kopeikina et al., 2011). Moreover, the selective impairment of calcium signaling (reduced *Calm2*, *Itpr1*, *Atp2a2*) and phagocytosis (reduced V-ATPase subunits) in A152T-AAV mice, coupled with increased apoptotic signaling (elevated *Casp3*) (Figure 5), points to a model where soluble tau species interfere with fundamental cellular processes essential for neuronal survival and protein clearance. This constellation of effects may create a feed-forward cycle exacerbating tau pathology and neurodegeneration, potentially explaining the neuronal loss previously observed both in this model as well as other models expressing A152T mutant tau (Carlomagno et al., 2019; Maeda et al., 2016; Decker et al., 2016).

Limitations of the current study that need to be considered include the modest sample size, as well as the inherent constraints of mouse models in fully representing the complexity of human tauopathies that develop over decades. Moreover, an important consideration is that the use of AAV to model tauopathy limits the directly affected cell types (in terms of exogenous mutant tau expression) to those that are transduced by AAV. For example, despite the use of a ubiquitous promoter (cytomegalovirus enhancer/chicken β-actin), published studies have shown that AAV1 exhibits poor microglial transduction efficiency following intracerebroventricular injection (Chakrabarty et al., 2013; Hammond et al., 2017). As such, differences observed in microglial signaling between the different tau models in the current report are independent of AAV-mediated mutant tau expression directly in microglia, but rather due to differences in microglial responses to P301L and A152T mutant tau expression in other cell types. This direct comparison of two different tau mutations (P301L and A152T) using identical AAV delivery methods, timeframes and analytical approaches provides a uniquely controlled evaluation of how different tau species affect cellular responses. This parallel design also allows us to attribute observed differences directly to the distinct properties of the tau species rather than to variations in experimental methodologies that often complicate cross-study comparisons. Another major strength of our study is the direct analysis of RNA transcripts rather than cDNA, eliminating potential amplification artifacts in gene expression profiling. Future research directions could include tau seeding studies comparing the propagation dynamics of fibrillar versus oligomeric tau, additional mouse models expressing specific tau species, and comparative analyses with human post-mortem tissue.

Taken together, our findings have important implications for understanding the pathogenesis of different tauopathies. The P301L-AAV model demonstrated that insoluble tau accumulation drives a strong microglial inflammatory response without substantial metabolic disruption, suggesting a direct response to tau fibrils rather than a consequence of cellular dysfunction. Conversely, the A152T-AAV model revealed that soluble tau species exert toxic effects through multiple mechanisms, including coordinated impairment of glucose metabolism, mitochondrial function, calcium signaling, and protein clearance pathways. Overall, this work demonstrates that different patterns of tau accumulation elicit fundamentally distinct glial responses and cellular pathologies, enhancing our understanding of how tau contributes to neurodegeneration and potentially informing more targeted therapeutic strategies based on predominant patterns of tau accumulation.

## Data Availability

The original contributions presented in the study are included in the article/Supplementary Material, further inquiries can be directed to the corresponding author/s.

## Abbreviations

AAV, Adeno-Associated Virus; CBD, Corticobasal degeneration; PSP, Progressive supranuclear palsy; PiD, Pick’s disease; AD, Alzheimer’s disease; SBT, Somatic brain transgenesis; FTLD-tau, Frontotemporal lobar degeneration with tau pathology; DAM, Disease-Associated Microglia; GFP, Green Fluorescent Protein; MGnD, Microglial neurodegenerative phenotype; ICV, Intracerebroventricularly; TCA, tricarboxylic acid; TNF, tumor necrosis factor; MCI, mild cognitive impairment.
